# Gut microbiome diversity influenced more by the Westernized dietary regime than the body mass index as assessed using effect size statistic

**DOI:** 10.1002/mbo3.476

**Published:** 2017-07-04

**Authors:** Shannon C. Davis, Jagjit S. Yadav, Stephanie D. Barrow, Boakai K. Robertson

**Affiliations:** ^1^ Microbiology Program Department of Biological Sciences College of Science, Technology, Engineering and Mathematics Alabama State University Montgomery AL USA; ^2^ Microbial Pathogenesis and Immunotoxicology Laboratory Department of Environmental Health College of Medicine University of Cincinnati Cincinnati OH USA

**Keywords:** body mass index, dysbiosis, effect size, gut microbiome, nextgen sequencing, obesity, overweight, Westernized diet

## Abstract

Human gut microbiome dysbiosis has been associated with the onset of metabolic diseases and disorders. However, the critical factors leading to dysbiosis are poorly understood. In this study, we provide increasing evidence of the association of diet type and body mass index (BMI) and how they relatively influence the taxonomic structure of the gut microbiota with respect to the causation of gut microbiome dysbiosis. The study included randomly selected Alabama residents (*n* = 81), including females (*n* = 45) and males (*n* = 36). The demographics data included age (33 ± 13.3 years), height (1.7 ± 0.11 meters), and weight (82.3 ± 20.6 kg). The mean BMI was 28.3 ± 7.01, equating to an overweight BMI category. A cross‐sectional case–control design encompassing the newly recognized effect size approach to bioinformatics analysis was used to analyze data from donated stool samples and accompanying nutrition surveys. We investigated the microbiome variations in the *Bacteroidetes‐Firmicutes* ratio relative to BMI, food categories, and dietary groups at stratified abundance percentages of <20%, 20%, 30%, 40%, 50%, 60%, and ≥70%. We further investigated variation in the *Firmicutes* and *Bacteroidetes* phyla composition (at the genus and species level) in relation to BMI, food categories, and dietary groups (Westernized or healthy). The Pearson Correlation coefficient as an indication of effect size across Alpha diversity indices was used to test the hypothesis (H_0_): increased BMI has greater effect on taxonomic diversity than Westernized diet type, (H_a_): increased BMI does not have a greater effect on taxonomic diversity than Westernized diet type. In conclusion, we rejected the (H_0_) as our results demonstrated that Westernized diet type had an effect size of 0.22 posing a greater impact upon the gut microbiota diversity than an increased BMI with an effect size of 0.16. This implied Westernized diet as a critical factor in causing dysbiosis as compared to an overweight or obese body mass index.

## 1 Introduction

Scientific investigations have demonstrated that the development of many metabolically based human diseases such as obesity, is associated with taxonomic changes occurring among the particular species of bacteria constituting the gut microbiota. This residential population permanently resides within the distal large intestines of all humans and other animals. Their primary role, among an array of other life sustaining biological functions, is that of a dietary energy extractor assisting the human host in gaining nutrients from the otherwise indigestible components of fresh, plant‐based foods (Diamant, Blaak, & de Vos, 2011; Kau, Ahern, Griffin, Goodman, & Gordon, 2011). The gut microbiota are highly susceptible to both biological and environmental influences. To varying degrees, several factors including the method of fetal delivery, neonate feeding, human genetics and disease, certain medical interventions and environmental exposures, such as having an abundant access to and the regular consumption of processed foods, are all known contributors to the taxonomic shifts within the microbiota populations. This occurrence is a disorder referred to as gut microbiome dysbiosis, and one that has been associated with the onset of many human diseases and disorders (Backhed, 2005; Cordain et al., 2005; Dalal & Chang, 2014; Davis, Barrow, Javan, & Robertson, 2015; Davis, Ogunbi, Ogunbi, & Robertson, 2015; De Filippo et al., 2010; Flint, Duncan, Scott, & Louis, 2014; Moreno‐Indias, Cardona, Tinahones, & Queipo‐Ortuño, 2014; Ramezani & Raj, 2013; Truswell, 2013; Xu & Knight, 2014).

Within the past decade, investigators have reported that the taxonomic diversity among the species constituting the gut microbiota as well as the ratio of two major bacterial phyla commonly abundant within the community, the Bacteroidetes and Firmicutes, is closely associated with both gut microbiota and human health. However, much information is still widely unknown in terms of gut microbiota health, including how a ‘healthy’ gut microbiome (e.g., the specific species as well as their expressed byproducts and genes) is characterized and alternately, how gut microbiome dysbiosis is related to human disease causation and vice versa. Human studies including overweight and obese participants have shown an association with a *decrease* in the abundance of Bacteroidetes, an *increase* in the abundance of Firmicutes, and with an overall *decrease* in the diversity of the gut microbiota population (Bäckhed et al., 2012; Clarke et al., 2012). Other investigations have provided no proof of these occurrences or have shown conflicting results (Duncan, Sadanand, Davachi, 2012). Therefore, there is a fundamental need for more taxonomic‐based studies investigating the structural characteristics of the gut microbiota in association with healthy and diseased (e.g., overweight, obese) participants as well as the incorporation of a systemic approach for evaluating various biological and environmental factors that impact the taxonomic profile and ultimately, the functionality of the gut microbiome (DiBaise, Frank, & Mathur, 2012; Walker et al., 2015).

While the *p*‐value is statistically informative, it does not measure the size or the magnitude of the effect between the factors being investigated and or compared to a disease state. The *effect size* (ES) calculation (SD_pooled_ = √((SD_1_^2^ + SD_2_^2^) ⁄ 2), does provide such statistically valuable information. Specifically within gut microbiome taxonomic‐based studies, ES is a measure of the distance in the vector of taxa frequencies (e.g., how far apart π_1_ and π_2_ are from each other). Understanding that the ES value allows for the universal comparisons across experiments, gut microbiome‐based studies are now rapidly shifting to include the statistic to measure the associations between participant demographics and health status, with the functionality of the gut microbiota and disease causation (Chen et al., 2016; Ravel et al., 2014; Song et al., 2016). It has been reported that future gut microbiome research will also include localized strategic collaborations among microbiologists, clinicians, bioinformaticians and the community as more human inclusive studies are needed to better understand the gut microbiota within their natural habitat; a concept referred to as *citizen science* (Dave, Higgins, Middha, & Rioux, 2012; Borel, 2014).

In the present study, we specifically aimed to investigate variation in the abundance of the Firmicutes and Bacteroidetes phyla composition at the genus and species level in relation to BMI, food categories, and dietary groups (Westernized or healthy). We also investigated microbiome variations in the Firmicutes:Bacteroidetes ratio relative to BMI, food categories, and dietary groups at stratified abundance percentages of <20%, 20%, 30%, 40%, 50%, 60%, and ≥70%. The Pearson Correlation coefficient as an indication of effect size across Alpha diversity indices was used to test the hypothesis (H_0_): increased BMI has greater effect on taxonomic diversity than Westernized diet type, (H_a_): increased BMI does not have a greater effect on taxonomic diversity than Westernized diet type.

## 2 Materials & Methods

### 2.1 Study ethics statement

This study was implemented within the state of Alabama and approved by the Alabama State University Institutional Review Board, approval number 2014CSMT002A. The study materials including dietary surveys and accompanying stool sample collection kits were randomly distributed among and collected from only Alabama residents, May 2015 through December 2015.

### 2.2 Study design

A cross‐sectional case–control study *design* was used to investigate study aims. Through this model, we were able to directly investigate known factors of gut microbiome dysbiosis causation to determine if a Westernized dietary regime had a greater association with lower gut microbiota diversity more so than the obese BMI group compared to nonobese and the healthy diet group (Yardley & Bishop, 2015).

### 2.3 Study population and sample size

Without regard to weight status, race, sex, or geographic location within the state, noninstitutionalized, otherwise healthy Alabama residents, ≥19 years of age, capable of understanding ‘informed consent’ on their own accord were randomly selected for study inclusion. The target sample size was determined based upon a fully parametric, bio‐statistical methodology derived from the Dirichlet‐multinomial distribution model recently presented by human microbiome researchers using a case–control study model similar to present study where researchers also incorporated the use of the *effect size* equation. As reported, to obtain a standard 5.0% significance level and a 99.99% power in accurately detecting a small effect size across our study groups, present study required a target sample size of at least fifty (*n* = 50) participants and at least ≥20,000 DNA sequencing reads per sample (La Rosa et al., 2012). Present study included a total study population of 81 participants and the taxonomic data were analyzed at ≥22,000 reads/sample.

### 2.4 Dietary surveys

Using a *blended survey format* allowed for the direct investigation into the specific foods (e.g., processed and fresh) being consumed by participants and the frequency of consumption as well as their BMI status (Yardley & Bishop, 2015). The specific questions that were used in the survey were formatted based upon nationally known food and health surveys including, (i) the Harvard University Health Professionals Follow‐up Study Questionnaire, (ii) the 2013–2014 Centers for Disease Control and Prevention National Health and Nutrition Examination Survey and (iii) the Dish‐based Semi‐quantitative Food Frequency Questionnaire for Assessment of Dietary Intakes (Centers for Disease Control and Prevention, 2015; Keshteli et al., 2014; Willett, 2014 **)**. Broad survey categories included questions relating to participant demographics, physical symptoms, eating behaviors, health status, and food consumption frequency. Descriptions of these categories are summarized in Table 1. To specifically assess the participant's overall diet type as well as the actual foods they consumed, the survey included a section for *24‐hr dietary recall* (e.g., all foods eaten the day before stool collection), a participant generated list of their *favorite foods* (e.g., consumed over a month), and an assessment of the *frequency of consumption* of predetermined processed and fresh foods that were consumed by the participant on a weekly basis.

**Table 1:** Summary of nutrition and health categories included in study survey

Question category (*n* = number of questions)	Description
Participant demographics (*n* = 10):	Age, race, sex, zip code, medical history, diet type
Food cravings (*n* = 5):	Frequency of experiencing fat, salty and sweet food cravings, overall frequency of cravings and experiencing moods that lead to cravings
Eating behaviors (*n* = 6):	Frequency of excessive eating, food addiction, craving and mood induced eating, visually induced eating.
Physical symptoms (*n* = 13):	Frequent abdominal cramping, bloat, constipation, diarrhea, hunger after eating, midday fatigue, regular bowel movement, high energy
Food consumption (*n* = 20):	24 hr dietary recall, listed favorite foods, frequency of consumption of selected fresh and processed foods
Participant opinions (*n* = 6):	Food access, food cost, preference in types of foods consumed, feeling of wellbeing

### 2.5 Metadata collection and transformation

The responses provided by the participants were numerically coded and transformed using a standard qualitative–quantitative approach (Bower, 2013; Nollet, 2004; Srnka & Koeszegi, 2014). The responses related to the questions of consumption frequency including (never, monthly, 1–2 Week, 3–4 Week, 5–6 Week, 7–8 Week, 9–10 Week, >11 Week) were coded as (0.05, 1.05, 20.00, 30.00, 50.00, 80.00, 90.00, 100.00), respectively.

**Figure 1:**
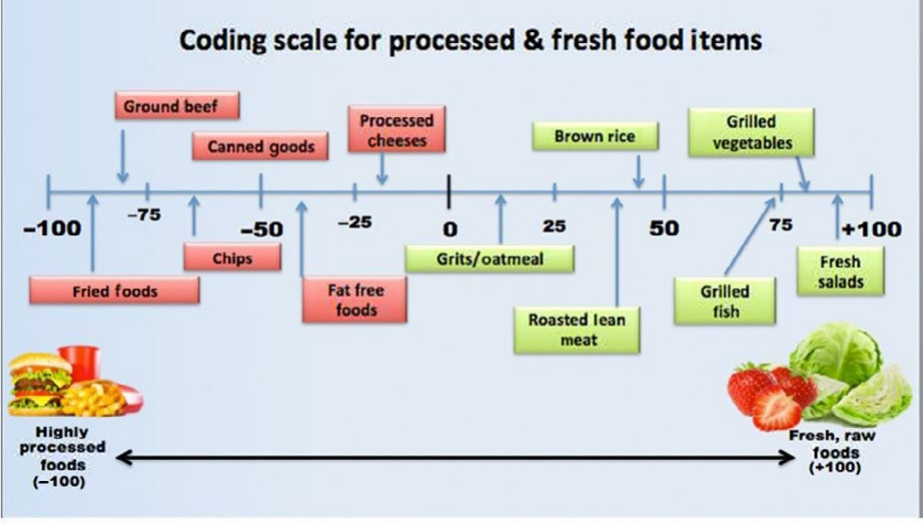
Degree of difference scale used for coding reported food items. Individual food items reported by the participant were first categorized as either processed (−) or fresh (+). Using 10‐point increments, the food was further categorized based upon the degree of processing or freshness by using known foods as comparative markers at −100 and +100 ends of the scale as shown on the schematic. Overall, using such scale provided a truer representation of the participants’ diet type compared to what they initially reported and it also allowed for assessing of the quality of foods as the negative and positive values representing the degree of food processing or freshness

A *degree of difference line scale* was utilized to numerically differentiate the food items reported by the participants within the 24‐hr diet and favorite foods survey sections (Davis, Barrow, et al., 2015; Davis, , Ogunbi, et al., 2015). As shown in Figure 1, a 100‐point scale was used to first categorize the individual food item as either processed (−100 scale) or fresh (+100 scale). Using the 10‐point increments within each side of the scale, the food was further categorized based upon the degree of processing or freshness by using known food markers at the −100 and +100‐point scale ends, (e.g., fast food bacon double cheeseburger = −100; raw fruit or water = +100). Overall, the degree of difference line scale allowed for a truer representation of the participants’ diet type compared to what they initially reported. This scale also allowed for assessing of the food *quality* with the negative and positive values representing the degree of food processing or freshness. Lastly, we also derived a *total food score* for each participant equating to the summation of the values for all food‐related survey categories. The highest positive total food score a participant could receive was 2650.00. This score implied that the participant only consumed a natural, fresh diet and that they did not consume any processed foods or sugared beverages. The total food scores for study population ranged from +1656.45 to −1116.50.

### 2.6 Stool samples collection and transport

Study participants were provided with an at‐home stool collection kit. Each kit contained a copy of our research brochure, an instruction sheet, informed consent, the dietary survey as well as the necessary accessories for the participant to carry out an aseptic stool sample collection. Fresh stool was collected using the Fisher Scientific Commode Specimen Collection System (Cat# 02‐544‐208). The collected sample was then placed into a Fisher Scientific C & S ParaPak (Culture & Sensitivity) Transport Vial (Cat# 23‐290‐147) (Fisher Scientific, 2015). Upon completion of sample collection, participants were instructed to place the vial into the liner bag contained within the Fisher Science Therapak^™^ Biological Substance Category B Ambient Shipping System (Cat# 22‐130‐025), and to then follow the instructions printed on the box for proper marking and sealing.

In accordance with the United States Postal Service, Mailing Standards‐Division 6.2 Infectious Substances, the Therapak containing the ParaPak C&S specimen vial along with the signed consent form and completed survey, were mailed in a premetered U.S. Priority Mail (2–3 day) enforced envelope directly from the participant's home to our laboratory (United States Postal Service, 2015). To further protect the identity of the participant, the outer U.S. Priority Mail envelope was preaddressed from our laboratory, as well as to our laboratory. After an initial quality assurance inspection, the signed consent forms were removed and stored separately from the dietary assessments and stool samples. Each sample and associated survey were assigned a corresponding laboratory ID code consisting of the consecutive number in line of intake at the laboratory, the day‐month of signing the consent form and the reported dietary group as either Westernized diet group, healthy diet group or obese group (e.g., ID: 113‐07/22‐WD).

## 3 Microbial DNA extraction and Illumina nextgen sequencing

### 3.1 Fecal microbial DNA extraction

Following the manufacturer's protocol, an aliquot (200 μl) of homogenized stool sample was used for microbial DNA extraction using a Zymo ZR Fecal DNA MiniPrep^™^ Isolation Kit (Cat# D6010). The DNA extract was immediately stored at −20°C (Zymo Inc, 2016). In this bead‐beating based protocol, an example of a lysed stool sample from a high fat diet showing thick lipid layer along the top (left sample), and another from a high fat diet with smaller lipid film, but containing a more mucoid consistency (right sample) are depicted within the supplemental material (see Attachment 1).

### 3.2 16S rRNA gene polymerase chain reaction (16S PCR)

**Figure 2:**
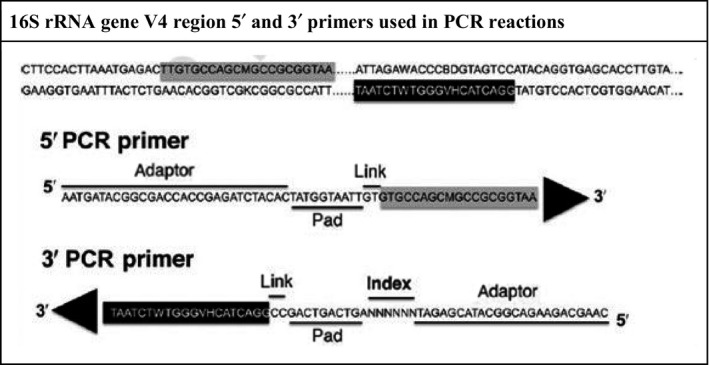
16S rRNA gene V4 region 5′ and 3′ primers used in PCR reactions. Highlighted in black and gray are the unique degenerate primer sequences used in the PCR reactions. As shown, 5′ and 3′ sequences contain an adaptor sequence, a link and pad sequence, and the degenerate sequences. The 3′ degenerate primer also contains a 6‐bp “barcoded” index sequence (NNNNNN) to distinguish the separate samples and study groups postsequencing reactions. Adapted from Kumar et al., 2014 with permission from author and publisher. All Rights Reserved.

The NEB LongAmp Taq PCR kit (Cat# E5200S) was used to carry out the PCR reactions. The unique degenerate barcoded primers used in the PCR reaction to amplify the V4 region of the 16S rRNA gene, are shown in Figure 2. The original primer stocks (50 nmol scale with a desalting purification) were sequentially diluted with 10 mmol/L Tris.Cl pH 8 first to 100 μmol/L and then to 10 μmol/L (working stock) for use in the PCR reactions. The 3′ degenerate primer also contained a (6‐bp) “barcoded” index sequence (NNNNNN) to distinguish the individual samples and study groups postsequencing. The PCR products were examined for specificity using 1.0% agarose/Tris‐borate‐EDTA gel electrophoresis and visualized by UV illumination and photography at (300‐380 bp). The Qiagen QIAquick Gel Extraction Kit was then used to purify the amplicons (Kumar et al., 2014).

### 3.3 DNA sequencing and bioinformatic analysis

The Illumina MiSeq 2000 nextgen sequencing platform was used for sequencing of the amplicons generated from the stool microbial DNA (Illumina, Inc., 2016). Quantitative Insights Into Microbial Ecology (QIIME), an open source bioinformatics pipeline, was used for the analysis of the sequenced DNA sequencing data. QIIME generated operational taxonomic units (OTUs) were deduced at standard 97.0% accuracy from the microbial DNA sequencing data and included both alpha and beta diversities.

## 4 Integrated analysis of metadata and microbial taxonomic information

### 4.1 Metadata scores

We analyzed the participant scores associated with categories of Fresh Food Consumption Frequency, Processed Food Consumption Frequency, 24‐hr Dietary Recall, Favorite Foods Report, and Total Food Score. Multiple sources of information such as these allowed for a deeper investigation into the relationships between the gut microbiota, diet, and disease including overweight, obesity, and gut microbiome dysbiosis.

### 4.2 Beta taxonomic indices

The abundance percentages for the *Bacteroidetes‐Firmicutes* (B‐F) phyla were determined for each participant at abundances of ≤19.99%, 20%, 30%, 40%, 50%, and >70%; the high and low cut‐offs were derived based upon the natural cut‐offs across the study population that allowed for meeting statistical analysis requirements. At each stratified percentage, we compared the B‐F ratio with factors of BMI and diet type along with the various food scores.

### 4.3 Alpha taxonomic indices

The second study aim included the incorporation of the effect size value determined based upon the results of the Pearson coefficient and traditional (p‐values). While we did not expect to find large effect sizes, the specific parameters for determination of the hypothesis were defined as, large effect values ranged from 0.4 to ≥0.8, and a small effect size values ranged from ≤0.2 to 0.3 (La Rosa et al., 2012).

### 4.4 Statistical analysis

Descriptive statistical analyses were conducted using IBM^®^ SPSS^®^ Statistics software package version 22, supplied through Information Technology, Department of Alabama State University.

## 5 Results

### 5.1 Study population demographics

Values for different demographic parameters (expressed as means and standard deviations) were as follows: age (33 ±13.3 years), height (1.7 ±0.11 meters), weight (82.3 ±20.6 kg), and BMI (28.3 ± 7.01), which equated to an overall *overweight* BMI. Sixty percent of the participants were of Caucasian race followed by African Americans with 28.0%, Hispanics 6.0%, mixed races 6.0%, and Korean with 3.0%. Assigned dietary categories included 53.0% participants in the Westernized Diet (normal) group, 16% in Westernized diet (obese) group, and 31.0% in the healthy diet group. A total of 49.1% were residents of Montgomery County. Other counties represented in present study included Autauga, Bullock, Chilton, Coffee, Cullman, Elmore, Geneva, Jefferson, Russell, St. Claire, Shelby, Tuscaloosa, Marion and Mobile. The study participants’ detailed demographic data are summarized in Table 2.

**Table 2:** Summarization of study participant demographics

Demographic	(*n*)%
Gender	
Male	(36) 44
Female	(45) 56
Age groups	
19–22	(18) 22
23–29	(28) 35
30–39	(9) 11
40–59	(18) 22
60–70	(8) 10
Dietary categories	
West diet group‐normal	(43) 53
West diet group‐obese	(13) 16
Healthy diet group	(25) 31
Race	
African American	(23) 28
Caucasian	(49) 60
Hispanic	(4) 6
Other (Mixed Race, Korean)	(5) 6
BMI categories	
Obese	(27) 33
Overweight	(27) 33
Normal‐underweight	(27) 33
Medical History	
Medical condition causing obesity (YES)	(6) 7
Mom obese (YES)	(54) 67
Fiber supplement (NO)	(69) 85

Overall, the study population was characterized by the following mean demographic parameters: mean height 1.7 ± 0.11 meters, mean weight 82.3 ± 20.62 kg, mean age 33 ± 13.3 years, and mean BMI 28.3 ± 7.01. Average BMI across the study population equated to overweight. Two participants were excluded due to recent antibiotic usage. No participant reported the use of probiotic; 85% participants used no fiber supplements.

### 5.2 Bacteroidetes:Firmicutes ratio

**Figure 3:**
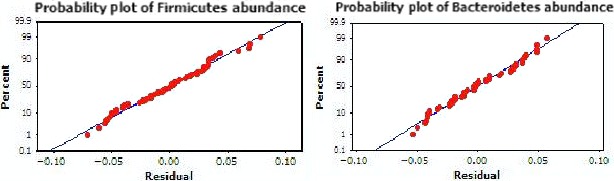
Distribution plots of the *Bacteroidetes* and *Firmicutes* phyla across the study population. Distribution plots of abundance percentages for B‐F phyla show both data sets are normally distributed with little variance total abundance across the study population. These findings suggest both B&F phyla are equally distributed across the population without regard to specific BMI category

Initial investigations into the abundance of the B‐F phyla across the total study population revealed a greater abundance of *Firmicutes* with a mean of 53.0 ± 0.18, compared to the *Bacteroidetes* (38.0 ± 0.18). This finding was expected as 53.0% of the study population consumed a Westernized Diet and overall, the study population had an average overweight BMI. Both factors of increased BMI and Westernized diet consumption have previously been associated with an increase in the *Firmicutes* (Kim, Gu, Lee, Joh, & Kim, 2012; Sonnenburg & Bäckhed, 2016). However, as shown in Figure 3 the abundance distribution plots of the B and F phyla provided evidence that both were overall normally distributed across the population.

An ANOVA analysis was used to compare the means of B‐F phyla at each of the abundance percentages ranging from ≤19.99% to 70.0%, in relationship to the normal‐underweight group and overweight‐obese group. Only two ANOVA tests were statistically significant as shown in Table 3. The first test showing significance was that of *Firmicutes* at the ≥40% midrange which was found among the normal‐underweight group (*F* = 8.73 df(1); *p* = .02). The second test showing statistically significant difference was also the *Firmicutes* at the lowest range (≤19.99%) among the overweight‐obese group (*F* = 6.26; df(1); *p* = .05).

**Table 3:** Variation in abundance percentages of *Bacteroidetes* and *Firmicutes* in relationship to BMI categories

	Normal‐underweight Group (*p*‐value)	Overweight‐obese group (*p*‐value**)**
≥70%	[*p* = .926]	[*p* = .735]
≥60%	[*p* = .490]	[*p* = .741]
≥50%	[*p* = .405]	[*p* = .759]
≥40%	[*p* = .016]^a^	[*p* = .753]
≥30%	[*p* = .617]	[*p* = .654]
≥20%	[*p* = .200]	[*p* = .567]
≤19.99%	[*p* = .311]	[*p* = .046]^a^

Individual ANOVA tests comparing the total percentages of B‐F abundance were used to determine variation in prevalence between the two phyla at stratified percentages in relation to BMI categories of normal‐underweight and overweight‐obese.

a Only two tests were of significance. The first among the normal‐underweight group [f(1)=8.73; *p* = .02], with a greater percentage of Firmicutes at the ≥40% range. The second test [f(1)=6.26; *p* = .05], showing greater percentage of Firmicutes at the ≤19.99% range among the overweight‐obese group.

ANOVA tests were used to further investigate the B‐F abundance at the various abundance percentages in relation to the survey categories including those of fresh food and processed food consumption scores, 24‐hr diet, and favorite food scores. At each abundance percentage (e.g., 70.0% range), an ANOVA was calculated for the B or F using all of the food scores for each category (e.g., processed, fresh foods). These results are summarized in Tables 5 and 6, showing the phylum with the higher mean within the table. Overall, each of the ANOVA tests was statistically significant (*p* = .001). In Table 4, the B‐F phyla are compared to fresh and processed foods. As shown, at the abundance percentage of ≥70.0%, the *Bacteroidetes* phylum had the higher total food score mean (−244.2 ± 127.4) in association with processed foods, and the *Firmicutes* were associated with the higher fresh food mean (382.7 ± 148.0). At the 60.0% range, the opposite occurred with the *Firmicutes* having the greater processed food score mean and *Bacteroidetes* having the greater fresh food score mean. Additionally, at the 40.0% and 50.0% abundance percentages the *Firmicutes* were found in association with higher fresh food means as well as within the lower abundance ranges of 20.0% to ≤19.99%. Alternately, the *Bacteroidetes* had the highest processed food means at abundance percentages of 50.0%, 40.0%, and 30.0%. While our initial findings showed that the *Firmicutes* were more prevalent across the study population, these results provided evidence that both *Firmicutes* and *Bacteroidetes* are associated with the consumption of processed and fresh foods and that the ratio of the phyla varies at different abundance percentages.

**Table 4:** *Bacteroidetes* and *Firmicutes* abundance in relationship to process and fresh food categories

	Bacteroidetes		Firmicutes	
	Fresh foods	Processed foods	Fresh foods	Processed foods
≥70%		[−244.2 ± 127.4]^a^	[382.7 ± 148.0]	
≥60%	[421.9 ± 250.3]			[−277.7 ± 215.2]
≥50%		[−354.9 ± 218.0]	[383.3 ± 154.9]	
≥40%		[−286.4 ± 223.4]	[309.8 ± 142.7]	
≥30%	[402.1 ± 160.8]	[−333.5 ± 250.8]		
≥20%	[419.9 ± 148.8]			[−335.5 ± 184.3]
≤19.99%	[299.0 ± 71.8]			[−263.6 ± 159.8]

a Values represent the mean and SD of the total food score at the stratified abundance percentage of the *Bacteroidetes* or *Firmicutes*. ANOVA test for both the B‐F groups was significant (*p* = .001). Overall, results suggest that despite the initial finding of an overall greater prevalence of *Firmicutes* across the study population, there is also evidence of association between the presence of *Bacteroidetes* and the consumption of both processed and fresh foods more so than the *Firmicutes*.

The continued ANOVA results showing the means of the B‐F phyla in relation to the 24‐hr diet and favorite food scores are summarized in Table 5. Each test was statistically significant at *p* = .001. The highest available food score available across the related survey categories was 2650.00. The value implies that all foods consumed by the individual were fresh and that they did not consume any processed foods or sugared beverages. By using this parameter, one can determine the degree of food processing or freshness of the foods reported in the 24‐hr diet and favorite foods categories by evaluation of the means associated with each food category. Overall, the means at each of abundance percentages were low, which implied the consumption of processed foods. As shown in Table 5, at the 50.0% to 70.0% range the *Bacteroidete*s were associated with the highest negative means in the 24‐hr diet category and despite the *Firmicutes* being associated with higher positive means at the same abundance range within the favorite foods category, the means are very low in terms of the total available food score (2650.00). At the midrange and lowest abundances ranging from 30.0% to <19.99%, the *Firmicutes* were associated with lower (negative) means, which again implies processed food consumption.

**Table 5:** *Bacteroidetes* and *Firmicutes* abundance in relationship to 24‐hr diet and favorite foods categories

	Bacteroidetes		Firmicutes	
	24‐hr diet	Favorite foods	24‐hr diet	Favorite foods
≥70%	[−139.1 ± 311.3]^a^			[72.6 ± 345.0]
≥60%	[−133.2 ± 202.0]			[42.0 ± 159.6]
≥50%	[−164.0 ± 282.3]			[21.0 ± 250.0]
≥40%		[−35.5 ± 153.0]	[−135.0 ± 307.8]	
≥30%		[104.5 ± 270.4]	[−229.8 ± 238.2]	
≥20%	[−42.2 ± 317.3]			[92.5 ± 151.2]
≤19.99%			[−186.2 ± 381.8]	[15.8 ± 379.9]

ANOVA tests of the B‐F abundance percentages across the 24‐hr Diet and Favorite Foods categories were significant [*p* = .001].

a Values represent the mean and SD of the total food scores within the category. At the 50.0% to 70.0% range, the *Bacteroidete*s were associated with the higher negative means and despite the *Firmicutes* being associated with higher positive means at the same abundance range, the means are very low (representing consumption of processed foods) in terms of the total available food score (2650.00). At the midrange and lowest abundances ranging from 30.0% to 19%, the *Firmicutes* were associated with lower (negative) means, which again implies processed food consumption. Overall, the *Bacteroidete*s were associated with the highest negative means at greater abundances.

### 5.3 Gut microbiome genus/species level diversity

Investigations into the B and F at the genus and species level in relation to food type (e.g., fresh or processed) and BMI categories (e.g., normal, overweight, obese) are summarized in Table 6 Using QIIME‐generated abundance data, the most prevalent gut microbiome species in association with each BMI group and food type along with the related total food scores were determined for each of the categories. As shown within the table, there were four phyla including the *Bacteroidetes, Firmicutes, Proteobacteria,* and *Actinobacteria* found among the most abundant microbial groups in relation to food type and BMI group. The specific genera and species per phyla were as follows: *Actinobacteria* (*Collinsella aerofaciens*), *Bacteroidetes* (*Bacteroides plebeius, Odoribacter sp*.)*, Firmicutes* (*Blautia obeum, Acidaminococcus sp., Catenibacterium sp., Dialister sp*.)*,* and the *Proteobacteria* (*Succinivibrio sp., Proteus sp*.). Closer investigations revealed that the abundance was tied between the obese BMI and processed food groups. For example, the *Collinsella aerofaciens* were abundant among the obese BMI and within processed food category equating in both groups to a total of 6.5% of the gut microbiota population of the individuals in the group. However, as indicated by the high negative total food scores within the BMI weight groups, it is apparent the species has a greater relationship with processed food consumption than with an increased BMI. Considering each species in the same manner, collectively the evidence further suggests that the Westernized diet type drives gut microbiota species selection more so than an obese BMI. These findings suggest that the previously known associations between increased *Firmicutes* and an increased BMI, may be occurring because the overweight or obese person is consuming a processed to highly processed diet type as found among this study population.

**Table 6:** Most abundant species in relation to BMI and food type

	↑ (%) Top 25 category	BMI, Abundance % & total food score
Actinobacteria		
*Collinsella aerofaciens*	6.5% [OB‐PF]^a^	OB [6.5; ‐1116.50], OW [5.10; −477.35], NW [2.1; −289.80]
Bacteroidetes		
Odoribacter sp.	9.3% [OB‐PF]^a^	OB [9.3; 22.15], OW [NA], NW [1.2; ‐289.80]
*Bacteroides plebeius*	17.2% [OB‐PF]^a^	OB [17.2; −289.23], OW [3.6; 1142.75], NW [1.9; −63.96]
Firmicutes		
Acidaminococcus sp	8.5% [NW‐PF]^a^	OB [4.5; 399.56], OW [5.3; 249.40], NW [8.5; −900.72]
Catenibacterium sp.	4.8% [OW]^a^	OB [2.3; −731.82], OW [4.8; 409.39], NW [2.1; −63.96]
Dialister sp.	24.3% [OB‐PF]^a^	OB [24.3; −1116.50], OW [5.1; −477.35], NW [4.8; −124.17]
*Blautia obeum*	8.4% [OB‐PF]^a^	OB [8.4; −620.95], OW [2.8; 1025.65], NW [2.2; 924.53]
Proteobacteria		
Succinivibrio sp.	15.0% [OW‐FF]^a^	OB [NA], OW [15.0; 601.10], NW [10.9; 169.70]
Proteus sp.	13.90% [NW‐FF]^a^	OB [2.3; −731.82], OW [4.8; 409.39], NW [13.9; 767.85]

a Denotes equal abundance percentages between the two survey categories. Overall, there is indication of Westernized diet type driving selection more so than obese BMI. As indicated by the high negative total food scores within the BMI weight groups, it is apparent the species has a greater relationship with processed food consumption than with an increased BMI.

**Figure 4:**
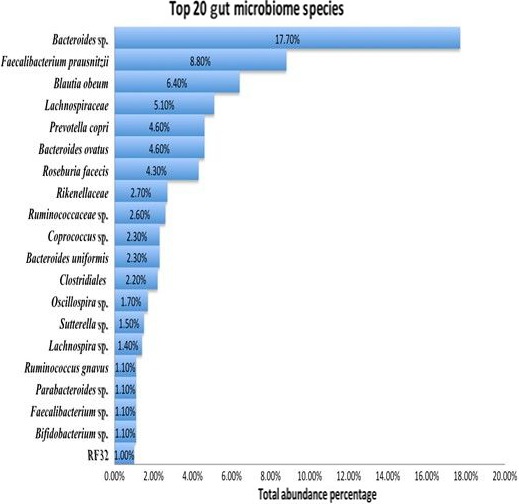
Top 20 gut microbiome species across total study population. The most abundant gut microbiome species (identifiable at species‐, genus‐, family‐, or order‐ level) found across the study population with the majority from the *Bacteroidetes* and *Firmicutes* phyla. As shown, the most abundant gut microbe found was the *Bacteroides spp*. at 18%, followed by *Faecalibacterium prausnitzii* at 9.0%

Figure 4 provides an overview of the most abundant gut microbiome species (identifiable at species‐, genus‐, family‐, or order‐ level) found across the study population. As shown, the majority of the species are from the *Bacteroidetes* and *Firmicutes* phyla. However, at that genus/species level, the most abundant gut microbe type found was the *Bacteroidetes* genus *Bacteroides* at 18%, followed by the *Firmicutes* species *Faecalibacterium prausnitzii* at 9.0%. The other species are shown within Figure 4.

Albeit, deeper participant level investigations into the top five species provided further evidence that diet type is a major factor contributing to defining the taxonomic characteristics of the gut microbiota. The findings are summarized within Table 7 with each of the organisms cross referenced to the top three participants they are found in, along with the participants’ BMI category and their total food score. As shown, the *Bacteroides* constituted a total of 50.9% of the gut microbiota of an overweight (OW) individual who had a total food score of −70.0. The genus also constituted 40.3% of an obese OB participants’ microbiota who had a total food score of −70.0 and 38.3% of the gut microbiota of an OW person with a food score of 262.85. The other four species are represented in the same manner within the table. While the obese (OB) and OW BMIs predominated the weight groups within the top genus/species, the OW and OB participants also had low total food scores implying that they consumed processed to highly processed foods on a daily basis.

**Table 7:** Percentage of the most abundant microbial species shown in relation to the top three participants and their BMI and total food scores

	% of the microbe constituting the gut microbiota of participant	BMI group	Total food score
*Bacteroides sp.* a	50.9	OW	−70.0
	40.3	OB	−70.0
	38.3	OW	262.85
*Faecalibacterium prausnitzii*	22.0	OW	−284.85
	21.1	OW	485.0
	21.0	OW	−407.8
*Blautia sp*.	20.2	OW	−199.8
	19.2	OW	−1425.0
	17.1	NW	−38.20
*Lachnospiraceae species*	15.0	OB	−550.0
	11.5	OW	−1424.95
	11.0	OB	−58.75
*Prevotella copri*	56.2	NW	−50.0
	43.0	NW	−829.35
	42.0	NW	649.0

a The *Bacteroidetes* genus *Bacteroides* represented the most abundant gut microbiota group with a total abundance of 18.0% across the study population. The second most abundant gut microbe species *F. prausnitzii* constituted 9.0%, followed by the genus *Blautia* at 6.4%, the family *Lachnospiraceae* at 5.1%, and the species *P. copr*i at 4.6% abundance. As shown, the *Bacteroides* was the most abundant genus within the gut microbiota population of an overweight (OW) individual constituting a total of 50.9% of the individuals’ gut microbiota with a total food score of −70.0. The genus also constituted 40.3% of an obese (OB) participant microbiota who had a total food score of −70.0 and 38.3% of the gut microbiota of an OW with food score of 262.85. Despite the *Firmicutes* phylum being the most predominate across the study population, at the genus level the *Bacteroides* prevailed.

**Figure 5:**
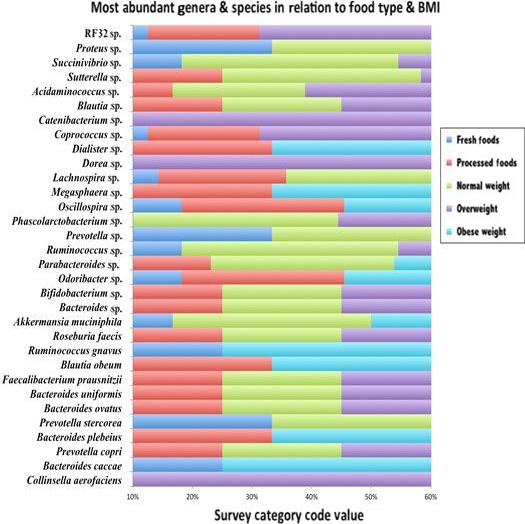
Distribution of the most abundant species associated with food types & BMI categories. The top species are represented along *Y*‐axis and the survey category code along *X*‐axis; [fresh food = (blue), processed food = (red), normal weight = (green), overweight = (purple) and obese = (teal blue)]. As there were more species found (among the Top 25) for the food types as indicated by red bars (processed foods) and blue bars (fresh foods) compared to increased BMI as indicated by purple bars (overweight BMI) and teal blue bars (obese BMI), we surmised again that diet type more so than BMI is driving the gut microbiota species selection within this study population

Figure 5 provides a graphic representation of the distribution of the most abundant species of the phyla *Actinobacteria*,* Bacteroidetes, Firmicutes, Proteobacteria, and Verrucomicrobia* in relationship to food types and BMI categories. The *fresh food group* is represented by blue bars (10–20 range on *X*‐axis)*, processed food group* by red bars (20–30 range), *normal weight* BMI by green bars (30–40 range), *overweight group* by purple bars (40–50 range), and the *obese group* by teal blue bars (50–70 range). The top abundance percentages ranged from 1.0% to 58.6%. While the majority of the identified genera/species were from the *Bacteroidetes* and *Firmicutes* phyla, some were also from the other three major phyla *Actinobacteria*,* Proteobacteria,* and *Verrucomicrobia*. The most abundant genera (with unidentifiable species) were *Actinobacteria* (*Bifidobacterium sp*.), *Bacteroidetes* (*Bacteroides sp., Odoribacter sp., Parabacteroides sp*., *Prevotella sp*.)*, Firmicutes* (*Phascolarctobacterium sp., Oscillospira sp., Megasphaera sp., Lachnospira sp., Dorea sp., Dialister sp., Coprococcus sp., Catenibacterium sp., Ruminococcus sp., Blautia sp., Acidaminococcus sp*.), and *Proteobacteria* (*RF32, Sutterella sp., Succinivibrio sp., Proteus sp*.). The most abundant identifiable species were as follows *Actinobacteria* (*Collinsella aerofaciens*)*, Bacteroidetes* (*Bacteroides caccae, Prevotella copri, Bacteroides plebeius, Prevotella stercorea, Bacteroides ovatus, Bacteroides uniformis*)*, Firmicutes* (*Faecalibacterium prausnitzii, Blautia obeum, Ruminococcus gnavus, Roseburia faecis*)*,* and *Verrucomicrobia* (*Akkermansia muciniphila*).

Viewing Figure 5, overall there were more genera/species found among the processed food group (represented by red bars), compared to the obese BMI (in teal blue bars) and the majority of the genera/species found specifically among the obese BMI were also found among the processed food group. The processed food category had a greater association with species of the phylum *Firmicutes* including *Blautia sp., Coprococcus sp., Dialister sp., Blautia obeum, Megasphaera sp., Lachnospira sp., Oscillospira sp., Roseburia sp.,* and *Faecalibacterium prausnitzii*. Within the obese BMI, there was an association with the species of the *Firmicutes* phylum namely, *Dialister sp., Ruminococcus gnavus, Blautia obeum Megasphaera sp., Oscillospira sp.,* and the phylum *Verrucomicrobia* species namely *Akkermansia muciniphila*. However, as there were more red bars (processed foods) and blue bars (fresh foods) compared to purple bars (overweight BMI) and teal blue bars (obese BMI) found in relation to certain gut microbiota species, we surmised again that dietary regime more so than BMI is driving the gut microbiota species selection within this study population.

### 5.4 Alpha & beta diversity indices

**Figure 6:**
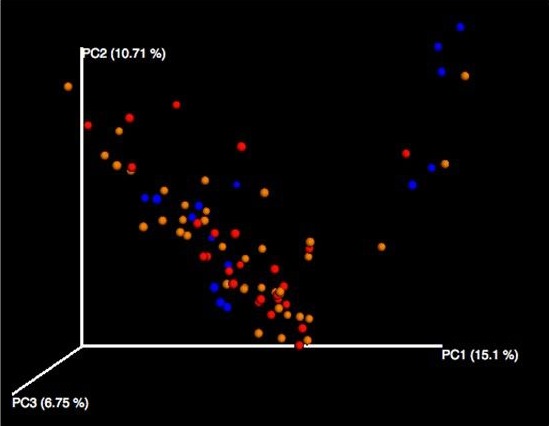
Bray–Curtis test results for the dietary groups. Bray–Curtis Test result revealed statistically significant [*p* < .05] dissimilarity across the dietary groups. The healthy diet group is shown in red, the West diet‐obese group shown in blue and the West diet‐normal weight group shown in orange. Considering the cluster pattern, it is initially indicated that diet type, more so than BMI, may possibly be driving the diversity of gut microbiota populations as the Westernized diet groups are aligning along with the healthy group (red) and there is no distinct cluttering by BMI

Bray–Curtis Test results associated with gut microbiota beta diversity are visualized within the principal coordinates analysis plot shown in Figure 6. These revealed there was statistically significant (*p* = .05) taxonomic dissimilarity across the dietary groups (healthy diet group, Westernized diet‐normal, and Westernized diet‐obese). Considering the cluster pattern however, it is indicated that the samples are grouping in relation to diet type as they are aligning along with the healthy diet group (red dots) and there is no distinct cluttering by BMI. This finding provided further indication that diet type may be driving the diversity of gut microbiota populations more so than an increased BMI.

**Figure 7:**
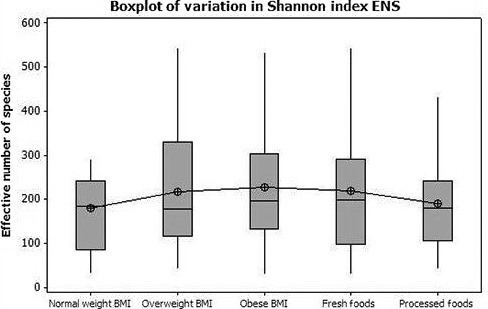
Variation in Shannon Index ENS values across BMI categories and food types. Shannon Index ENS means and SD per category were normal BMI (179.9 ± 103.1), overweight BMI (218.1 ± 134.2), obese BMI (228.2 ± 134.1), Total Food Scores‐ *Fresh* (220.0 ± 134.2), and Total Food Scores‐*Processed* (190.2 ± 109.7). No statistical variation across groups was found with a *p* = .53. However, as shown the obese BMI was associated with an increase in ENS and processed foods with a decrease

An ANOVA test using the means of the Alpha diversity Shannon Index ENS (effective number of species) associated with processed food, fresh food, and BMI categories did not reveal any statistical difference between the groups (*p* = .53). The highest ENS mean value was found in association with the obese BMI (228.2 ± 134.1). ENS values for the other categories included the normal BMI (179.9 ± 103.1), overweight BMI (218.1 ± 134.), fresh food group (220.0 ± 134.2), and processed food group (90.2 ± 109.7). As shown in Figure 7 the ENS increases with an obese BMI and decreases with the consumption of processed foods.

### 5.5 Effect size‐based hypothesis testing

Here, effect size (ES) was used to test the proposed hypothesis. To accomplish this, Pearson's Correlation statistics was performed for each alpha diversity matrix including those of the Shannon Index, Simpson's Index of Diversity, Chao1, and the whole tree diversity (Meehan & Beiko, 2014). These results are summarized in Table 8. As shown, no test resulted in a *p*‐value of any statistical significance with the values ranging from 0.06 to 0.74. However, there was variation found between correlations in terms of ES, ranging from 0.04 to 0.23. The effect size of the total food score, fresh foods, processed foods, 24‐hr diet, and favorite foods survey categories ranged from 0.16 to 0.31 with a total average of 0.22. The average effect size of obese and overweight BMI equated to 0.16. Considering the study hypothesis, we failed to accept the (H_0_), and concluded that the processed food type with an ES of 0.22 had a greater effect upon the overall diversity of the gut microbiota than an increased BMI with an average ES of 0.16. Through alpha and beta taxonomic investigations, we also demonstrated that the total abundance percentage chosen by an investigator could potentially influence the interpretation of their findings. We also demonstrated that using just one abundance percentage value without incorporation of metadata might not truly represent the nature of the B‐F ratio within the population or individuals being investigated.

**Table 8:** Effect sizes of correlations between BMI, Westernized diet, and Alpha diversity indices

	Shannon ENS		Simpson diversity		Chao1		Whole tree		
	Effect Size	*p* ‐Value	Effect Size	*p* ‐Value	Effect Size	*p* ‐Value	Effect Size	*p* ‐Value	Average Effect Size
BMI	−0.10	.21	0.15	.22	0.06	.53	−0.09	.44	0.16
Total food	0.20	.10	−0.14	.23	0.10	.43	0.14	.24	0.16
Fresh food	0.22	.06	−0.20	.1	0.08	.51	0.09	.50	0.17
Processed	0.21	.08	−0.18	.14	0.12	.33	0.07	.60	0.17
24 hr	0.08	.51	0.04	.73	0.05	.70	0.14	.25	0.30
Fav foods	0.22	.06	0.04	.74	0.04	.74	0.07	.56	0.31

Pearson's Correlation statistics was performed for each alpha diversity matrix to determine the correlation between increased BMI, food scores, and decreased ENS. As indicated, no test resulted in a *p* ‐value of any statistical significance with the values ranging from 0.06 to 0.74. However, there was variation found between correlations in terms of effect size, ranging from 0.04 to 0.23.

**Figure 8:**
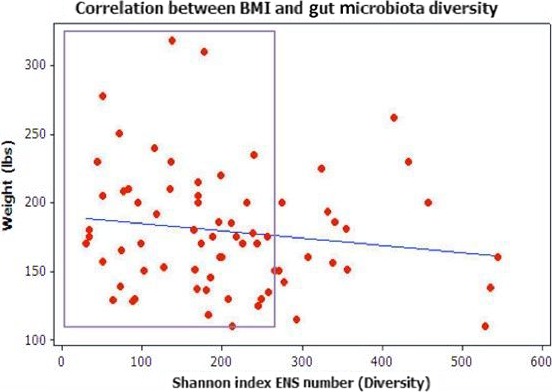
Pearson correlation between increased BMI and increased Shannon Index ENS. Pearson Correlation [*p* = .213]. Even though the test *p*‐value was not statistically significant, the cluster pattern of the results provided valuable evidence of the relationship between BMI and ENS. As highlighted within the box outline, the lowest ENS diversity (≈40 to 250 ENS) is associated with the lowest and highest weights. These findings suggest that a factor other than weight (BMI) could be driving total population diversity of the gut microbiota

The Pearson correlation results of the association between Shannon Index ENS (*X*‐axis) and BMI (*Y*‐axis) are shown in Figure 8. While the highest diversity (>500 ENS) is found at lower weights, there are also obese and normal weight individuals with high ENS. Highlighted within the box outline, the lowest diversity (≤250 ENS) is also associated with a range of weights from the highest and lowest among the study population.

**Figure 9:**
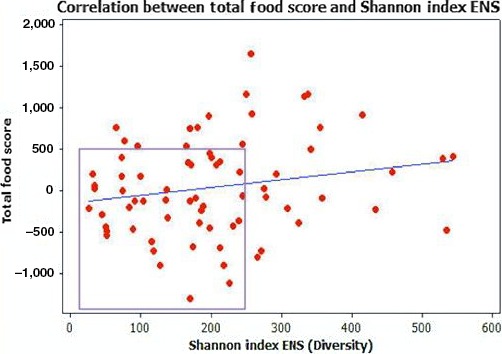
Pearson correlation between total food scores and Shannon Index ENS. Pearson Correlation [*p* = .10]. As highlighted, there is more clustering around a negative food score and lower ENS. While there are positive food scores associated with lower ENS (≤350), the fresh food scores are primarily low which indicated the consumption of minimally processed foods and within this particular study population, the higher fresh food scores (>1000) are not indicative of a completely fresh diet type

The Pearson correlation between a decreased total food score and Shannon Index ENS was not statistically significant (*p* = .10), as shown in Figure 9. As highlighted within the box, there is more clustering around the lowest negative food scores and lower ENS. While there are positive food scores associated with lower ENS (≤350), the fresh food scores are primarily low indicating the consumption of minimally processed foods.

**Figure 10:**
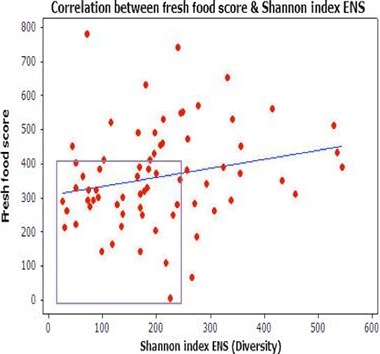
Pearson correlation between frequency of fresh food consumption and Shannon Index ENSPearson Correlation [*p* = .06]. The fresh food consumption scores shown along *Y*‐axis are low indicating low frequency of consumption of fresh, whole foods. As highlighted, the lower ENS of ≤250 is associated with lower consumption of fresh food. Alternately, the higher ENS is associated with an increased consumption of fresh, whole foods

**Figure 11:**
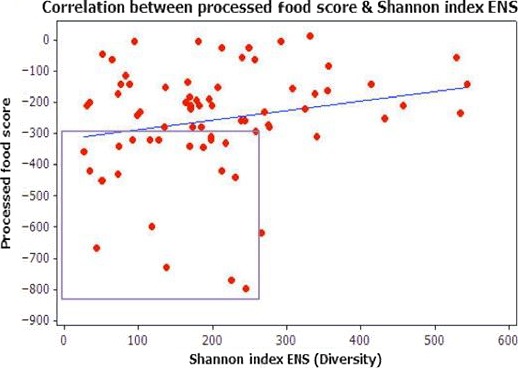
Pearson Correlation between frequency of processed food consumption and Shannon Index ENS. **Pearson Correlation [** *p* **= .077].** As shown within the boxed area, frequent processed food consumption indicated by the low negative values ranging from ‐350 to ‐875 along the *Y*‐axis, is associated with lower ENS (≤250).

As highlighted within Figure 10, the fresh foods score is shown along the *Y*‐axis. As highlighted within the boxed area, the lower ENS (≤250) is again associated with less frequent fresh foods consumption. As shown within the boxed area of Figure 11, there is greater clustering around lower ENS and increased consumption of highly processed foods. As demonstrated between both of these tests, the higher ENS or microbial diversity is associated with less consumption of processed foods and an increased consumption of fresh, whole foods.

**Figure 12:**
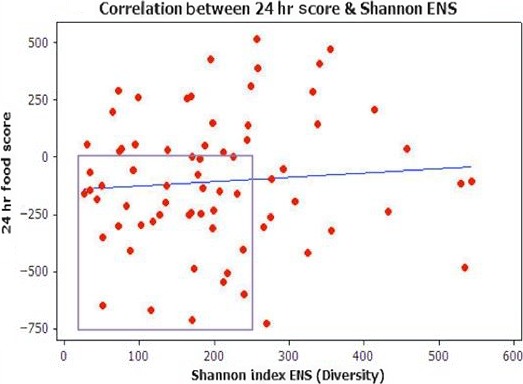
Pearson correlation between 24‐hr diet and Shannon Index effective number of species. Pearson Correlation [0.079; *p* = .507]. As indicated by the area highlighted by the outline, the lowest effective number of species is associated with the consumption of processed foods. To gain a perspective of how fresh the foods were in positive range, the highest obtainable fresh food score within present study (e.g., implying only fresh, whole foods and no processed foods are consumed) was 2650.00

**Figure 13:**
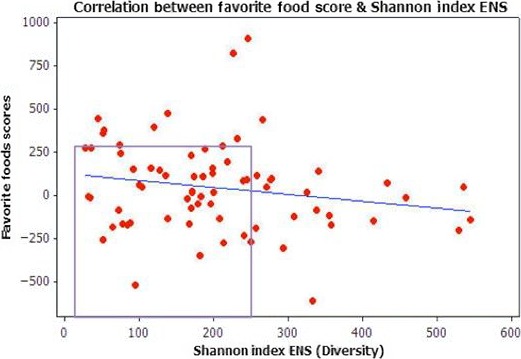
Pearson correlation between favorite foods and Shannon Index effective number of species. Pearson Correlation [*p* = .06]. As other results have shown, the lowest ENS has a greater association with the consumption of processed foods than with fresher foods. This is further evident considering that the highest food score here of 990 indicates the consumption of more minimally processed foods than fresh, whole foods as the highest obtainable fresh food score across the food categories (>2000). The highest obtainable fresh food score within present study was 2650.00 implying only fresh, whole foods and no processed foods are consumed

Pearson's correlations between the 24‐hr diet, favorite food categories, and Shannon Index ENS are shown in Figures 12, 13. The negative and positive values along the *Y*‐axis in both figures are indicative of the degree of food processing. To gain a perspective of how fresh the foods were in positive range, the highest obtainable fresh food score within present study (e.g., implying only fresh, whole foods, and no processed foods are consumed) was 2650.00. The highest fresh food score here was ≈950. As shown in Figure 12, the majority of participants consumed a minimally processed to highly processed meal 24 hr prior to the stool sample collection. Again, the lowest ENS is associated with the consumption of processed foods indicated by the area highlighted by the outline.

The same was found when investigating the association between ENS and favorites foods (e.g., consumed over a month) as depicted within Figure 13. As before, the lowest ENS was also found associated with processed food types. Collectively, we concluded that diet type has a greater effect upon the diversity of the gut microbiota than an increased BMI. Despite the Person's Correlation *p*‐values not being statistically significant, the effect size of processed food consumption upon the overall diversity of gut microbiota was greater than that of an obese BMI (See Table 9 for reference).

## 6 Discussion

It is increasingly being recognized that diet may be an important modulator of gut microbiota (Sonnenburg & Bäckhed, 2016). While perturbations associated with small dietary changes on a day‐to‐day basis may be reversible, long‐term dietary habits may cause more long‐lasting microbiota changes. For instance, long‐term intake of traditional fiber‐rich diets versus Westernized fat‐rich diet may have more irreversible changes, which may be transferred over generations (Sonnenburg et al., 2016). In this context, researchers are still elucidating the taxonomic characteristics of a ‘healthy’ gut microbiota as well as seeking to determine the primary factor, such as the regular consumption of processed food or having an obese BMI that contributes to gut microbiome dysbiosis and ultimately human disease causation (Sonnenburg et al., 2016).). Understanding that processed foods contain an excess amount of energy and that such foods have the propensity to cause inflammatory responses, the gut microbiota genus and species that were once thought to be ‘healthy’, may in fact be utilizing alternative metabolic pathways and or inducing negative host or gut microbiota inflammatory responses as the host diet evolves from a fresh, whole food diet to a Westernized diet type. Because gut microbiome dysbiosis is initially asymptomatic, these subtle changes within the gut microbiome go unnoticed by the human host and or their primary care physicians until metabolic disorders such as overweight and eventually obesity occur. While many future studies are necessary to make any definitive conclusions, our overall findings suggested that the specific dietary regime imparts a greater impact upon the B‐F ratio and ultimately the collective diversity of the gut microbiota population compared to an obese BMI (see Figure 5). Additionally, several findings gleaned from present study are of significance to the scientific community in fulfilling research gaps as our data have illustrated in some instances contradictory results from those previously published, and in other cases have helped move forward in understanding gut microbiome dysbiosis causation.

### 6.1 Species‐level investigations

As commonly found among individuals who consume a diet type rich in processed foods as well as in those with an increased BMI, our initial investigations revealed that the *Firmicutes* phylum predominated the study population at 52.4% compared to the *Bacteroidetes* at 39.0% (Sun & Chang, 2014). Contrary to published findings however, we found that the B and F phyla were evenly distributed across the study population. Additionally, we found the *Firmicutes* phylum was only statistically significant at the 40.0% abundance range among the normal weight BMI, and at the ≤19.99% abundance range within the obese BMI group (see Table 3) (Conlon & Bird, 2014). However at the genus/species level, the *Bacteroides spp*. prevailed with a total abundance of 18.0%. While a gut microbiota population consisting of predominately the *Bacteroidetes* phylum is considered to be healthy, here we found this phylum was most prevalent among OW or OB persons who consumed a Westernized diet type (see Table 7).

Fundamentally, all gut microbiota possess the ability to utilize various dietary‐derived substrates in the production of energy. Therefore, it was realized that the abundance of a species and or genus that is generally associated with a healthy gut microbiota population, could in fact be representative of an early symptom of gut microbiome dysbiosis instead of gut microbiome health. Processed foods contain high amounts of refined sugars, fats, and carbohydrates, chemical additives, and are low in natural plant fiber. These foods are also disproportionally balanced providing excess energy and little nutrition or no nutrition to both the gut microbiota and human host (Moss, 2013). Consumption of such diet type is directly associated with the onset of *gut microbiome endotoxemia* (Parekh, Arusi, Vinik, & Johnson, 2014). With the consumption of processed foods, gut microbiome endotoxemia is an *in vivo* inflammatory response occurring as the endotoxin lipopolysaccharide (LPS), releases from the cell walls of gram‐negative bacteria within the gut microbiota population (Darzi, Frost, & Robertson, 2011; Puertollano, Kolida, & Yaqoob, 2014; Rahat‐Rozenbloom, Fernandes, Gloor, & Wolever, 2014). With these understandings, the abundance of the genera/species shown in Figure 5 and Table 7 may in part be due to the increased dietary energy associated with the regular or over consumption of processed food products.

Specifically, the *Bacteroidetes* are exceptional dietary energy extractors with the capability to degrade both plant and refined carbohydrates by using carbohydrate‐processing enzymes (CAZymes) (Thomas et al., 2011). The higher abundance of the *Bacteroides spp*. found here, may be occurring because despite of the increased BMI the majority of study participants are consuming a diet of energy dense, carbohydrate rich processed foods, as indicated by their low total food scores. The *F. prausnitzii* is also thought to be a beneficial probiotic species as it stimulates the expression of the anti‐inflammatory cytokine interleukin 10 (Walker et al., 2010). The *F. prausnitzii* prevalence here could potentially be contributing to human health through its anti‐inflammatory capabilities as the consumption of a Westernized diet type induces the onset of *gut microbiome endotoxemia* which involves the activation of many host and or gut microbiota inflammatory responses (Bäckhed et al., 2012; Heinken et al., 2014; Tremaroli & Bäckhed, 2012). The genus *Blautia* is an acetogen bacterium that can utilize a variety of substrates including sugars or other organic substrates (Muller, 2003). Again, the individuals harboring this group all have negative food scores, with one participant having a total food score of −1425.0; this score indicating that the participant consumed highly processed foods and sugared beverages >11/week as their primary diet. The presence of *Blautia* could be an indication of increased consumption of refined sugars and or refined carbohydrates. In terms of human health, could also be an indication of gut microbiome dysbiosis.

The *Lachnospiraceae* family and *Prevotella copri* species have both been associated with human disease including overweight, obesity, and intestinal disorders (Meehan & Beiko, 2014). Nevertheless, species of the *Lachnospiraceae* family are also thought to be beneficial to the human host through the production of the short‐chain fatty acid butyric acid (as *butyrate*), reported to reduce chronic inflammatory conditions and the risk of colon cancer. While the *Lachnospiraceae* family has been associated with an obese BMI, here we found that the OW and OB participants in this group consumed highly processed foods as indicated by the total food scores of −1424.95, −550.0, and −58.75. The presence of *Lachnospiraceae* among these individuals may be indicative of the response to the increased consumption of highly processed foods resulting in a need by human and or gut microbiota to reduce the inflammatory conditions within the host (Butyrates, 2016). Lastly, it has been reported that the presence of the species *P*. *copri* is associated with a reduction in the prevalence of the *Bacteroides* and other beneficial gut microbiota as well as with the pathogenesis of human disease (Scher et al., 2013). Individuals in this group were normal weight (NW), all with low total food scores. The presence of species such as *P*. *copri*, could be indicative of early gut microbiome dysbiosis occurring as the individuals’ diet evolves to a Westernized diet type, but before systemic adiposity manifests as overweight and eventually obesity.

### 6.2 Hypothesis testing

Through the use of an effect size‐based hypothesis test investigation, we found concluding evidence suggesting that a Westernized dietary regime had a greater influence upon the taxonomic characteristics of the gut microbiota more so than an overweight or obese BMI (Chan, Estaki, & Gibson, 2013; Davenport et al., 2015; Festi et al., 2014). Initial Pearson's Correlation test *p*‐values did not provide any statistically significant results when comparing the association of a lower Shannon Index effective number of species with an increased (obese) BMI and processed food consumption. However, testing the study hypothesis we concluded that Westernized diet type with an effect size of 0.22 had a greater effect upon gut microbiota diversity than increased BMI with effect size of 0.16. Collectively, study findings suggested that using such parameter provides a more accurate representation in investigating bioinformatics and participant metadata as well as how some of these factors in turn, contribute to the causation of gut microbiome dysbiosis (Debelius, 2015; Greenhalgh, Meyer, Aagaard, & Wilmes, 2016; Morgan & Huttenhower, 2012; Shetty, Marathe, & Shouche, 2013).

### 6.3 Study limitations

As the Westernized dietary regime has become a common staple within the United States, there is increasing interest in understanding the role of diet in gut microbiome in human disease causation as well as a factor that can potentially impact future gut microbiome studies (Debelius, 2015). However, it is a challenge to identify appropriate comparison groups to investigate the effects of a Westernized dietary regime. The Amish population who reside within the small town of Faunsdale, Alabama (total town population of 95) is one promising group that has a unique lifestyle compared to the general U.S. population. While Amish group was reluctant to participate in this study, as shown in supplemental material Attachment 2, a family from that group was willing to discuss with us their traditions and lifestyle. As we spoke over lunch however, it was apparent that consumption of processed foods is inevitable even among groups who have traditionally refrained from consuming such foods (Cuyun Carter et al., 2011). In general, the larger the sample size, the better will be the interpretation of the reported effects. Importantly, to realize the power of both the effect size and *p*‐value in interpretation of reported effects, future extension of this initial study to a larger cohort, which may help improve the *p*‐values, may be pursued.

Another major challenge within present study was the fact that gut microbiome‐based studies have historically been technologically driven with stool sample data being gathered and analyzed without regard for inclusion of participant metadata and a standardized approach, making cross‐comparison and investigations into disease causation difficult. Therefore, it has been suggested that future gut microbiome studies capture more metadata that can be used to better understand the overall functionally of the gut microbiota and to assist scientists in gaining applicable bioinformatics data in terms of human health and or gut microbiome dysbiosis causation (Gevers, Pop, Schloss, & Huttenhower, 2012; Marchesi, 2014; Morgan & Huttenhower, 2012; Nguyen, Vieira‐Silva, Liston, & Raes, 2015). In this context, we tried to collect as much metadata as was feasible. However, the greatest limiting factor of gut microbiome studies still lies within overcoming the quantum physics observer effect theory, suggesting that the very act of observation affects the reality of that which is being observed. This theory applies to present study, as it is difficult to obtain a sample of the gut microbiome of an individual without the use of an invasive method that potentially disturbs the biofilms encasing gut microbiota that are attached to the intestinal walls. While obtaining a stool sample is noninvasive and easily carried out by the participant, researchers are ultimately getting only limited insight into the true dynamics of the core gut microbiota (Staley, 1997).

## 7 Conclusions

The study demonstrates that the Bacteroidetes‐Firmicutes abundance percentage chosen by an investigator could influence the overall interpretation of the findings, considering that both *Bacteroidetes* and *Firmicutes* phyla were associated with processed and fresh food consumption as well as with an increased BMI. To better understand which of these factors (e.g., processed food or obese BMI) is influencing the taxonomic structure of the gut microbiota, the use of the effect size statistic proved necessary. Overall, we also demonstrated that without the incorporation of participant metadata and universal effect size values, it is difficult to hone in upon what biological or environmental factors are actually impacting the gut microbiota. The use of these methodologies within this study led to the final conclusion that processed food consumption has a greater influence upon the gut microbiota structure and was associated with lower ENS diversity of the population, more so than an increased BMI (Ravel et al., 2014; Faloney et al., 2016; Forum 1, 2013). Additionally, present study has provided a deeper understanding of gut microbiome dysbiosis and human obesity causation and our findings along with subsequent future studies may set the stage for streamlining gut microbiome investigations through use of standardized approaches and methodologies; such developments would be serving as a springboard to reaching the next level of understanding of role of gut microbiome in causation of human disease.

## Conflict of Interest

The authors have no competing interests to declare.
