# Supplementary material for: Gut microbiome diversity influenced more by the Westernized dietary regime than the body mass index as assessed using effect size statistic
Source: Microbiologyopen. 2017 Jul 4;6(4):e00476. doi: 10.1002/mbo3.476 (PMC5552927; doi:10.1002/mbo3.476)

SUPPLIMENTAL MATERIAL

Supplemental Figure 1: Variable appearance of lysed stool sample from high fat diets

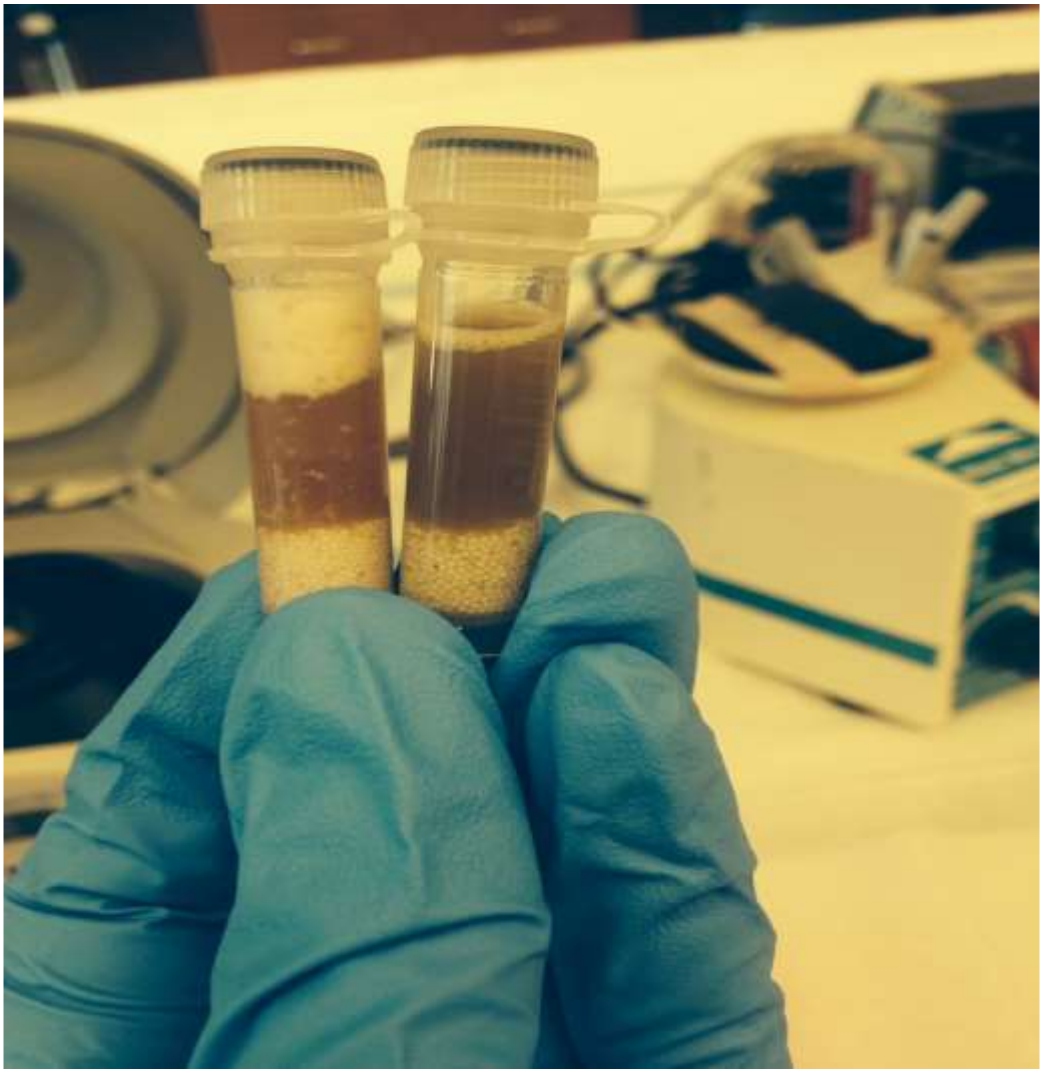

The thick lipid layer at the top of the sample on the left is indicative of consumption of a Westernized (high fat) diet type. While the sample on right is also from a high fat diet, there is a thinner lipid layer albeit with high viscosity. The bottom layer shows the cell-lysing micro beads. **Photo Credit: Shannon C. Davis [Canon Rebel Ti]**.

Supplemental Figure 2: Cafe in Faunsdale, Alabama

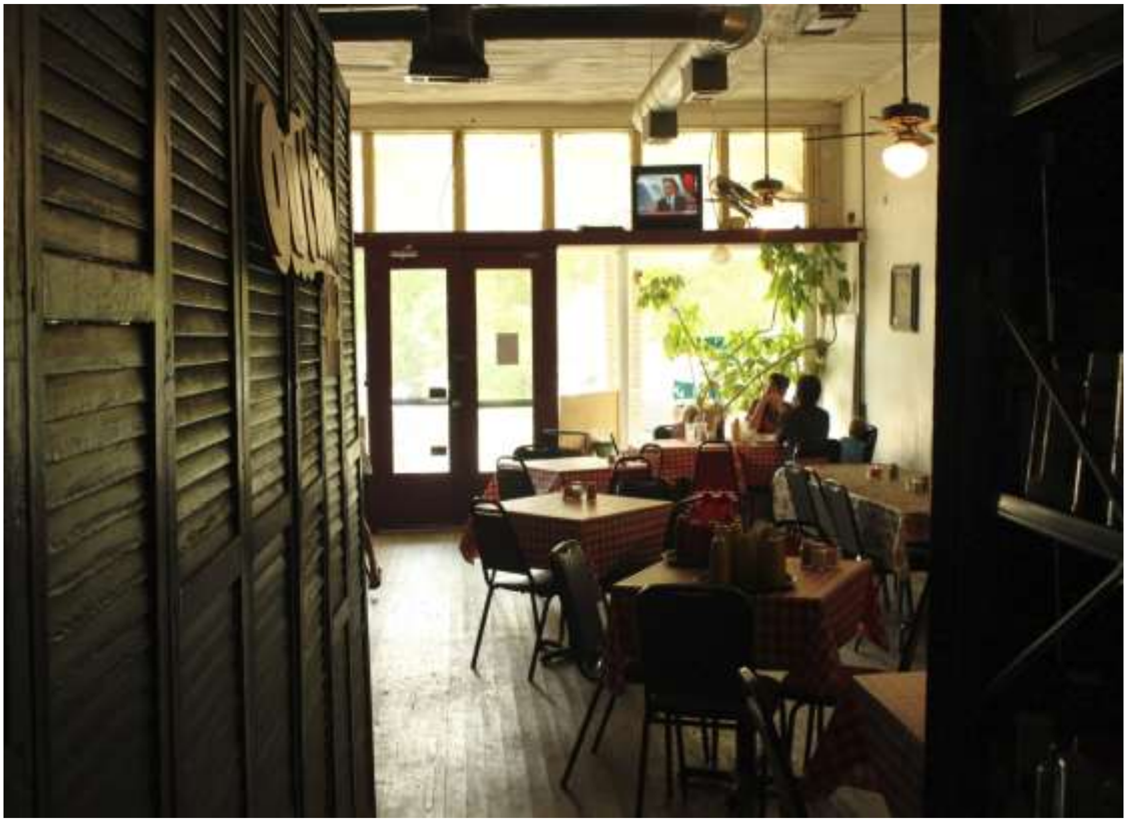

Meeting with an Amish family (top photo) at the Faunsdale Cafe (bottom right) in the small town of Faunsdale, Alabama. Total population in 2013 (n=95). The town is home to Cedar Crest Mennonite Church, the only Mennonite Church in the state as well as home to the second Amish population in Alabama outside of Greensboro [www.Al.Com]. Out of respect for the family, names and faces are withheld. While talking about their traditions over lunch, it became apparent that consumption of processed food within the United States is inevitable. The daily special (bottom left) fried catfish, French fries, a white bread slice, and side salad of ice burg lettuce with ranch dressing. **Photo Credit: Shannon C. Davis**.

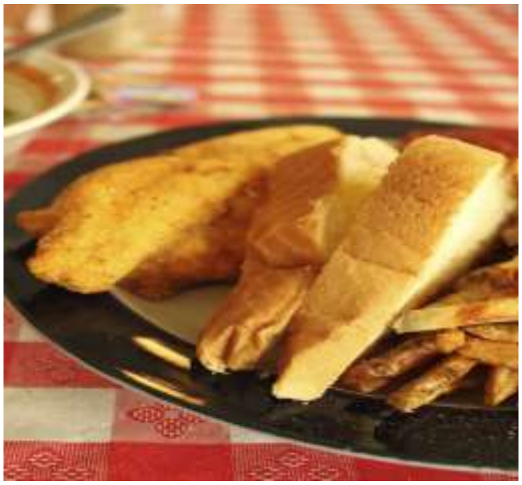

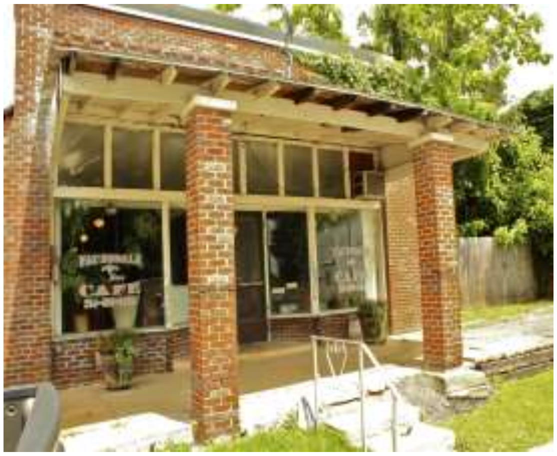

Supplement: Supplementary file 1 [file MBO3-6-na-s001.pdf]
